# Current Advances in Stimuli-Responsive Hydrogels as Smart Drug Delivery Carriers

**DOI:** 10.3390/gels9100838

**Published:** 2023-10-22

**Authors:** Yulong Zhang, Benjamin M. Wu

**Affiliations:** 1 Department of Mineralized Tissue Biology, The Forsyth Institute, Cambridge, MA 02140, USA; yzhang@forsyth.org; 2 Department of Orthopaedic Surgery, David Geffen School of Medicine, University of California, Los Angeles, Los Angeles, CA 90095, USA; 3 Department of Bioengineering, School of Engineering, University of California, Los Angeles, Los Angeles, CA 90095, USA; 4 School of Dentistry, University of California, Los Angeles, Los Angeles, CA 90095, USA

**Keywords:** smart hydrogel, nanogel, drug delivery, stimuli-responsive, pH, temperature, redox, light, glucose, enzyme, disease treatment, precision medicine

## Abstract

In recent years, significant advancements in the field of advanced materials and hydrogel engineering have enabled the design and fabrication of smart hydrogels and nanogels that exhibit sensitivity to specific signals or pathological conditions, leading to a wide range of applications in drug delivery and disease treatment. This comprehensive review aims to provide an in-depth analysis of the stimuli-responsive principles exhibited by smart hydrogels in response to various triggers, such as pH levels, temperature fluctuations, light exposure, redox conditions, or the presence of specific biomolecules. The functionality and performance characteristics of these hydrogels are highly influenced by both their constituent components and fabrication processes. Key design principles, their applications in disease treatments, challenges, and future prospects were also discussed. Overall, this review aims to contribute to the current understanding of gel-based drug delivery systems and stimulate further research in this rapidly evolving field.

## 1. Introduction

The discovery of the first hydrogel intended for biological use dates back to 1960 [1]. Since then, hydrogel systems have undergone significant improvements. This development has seen the transition of hydrogels from conventional “dumb” gels into “smart” gels capable of responding to unique external stimuli or environmental changes. In recent decades, smart hydrogels have received increasing attention in the field of drug delivery due to their stimuli-responsive properties; low invasiveness; ease of administration; and controlled, sustained drug release capabilities [2,3,4,5,6,7]. While conventional “dumb” gels can swell or shrink in response to osmotic pressure, their responsiveness is often inefficient, leading to imprecise drug release and limited control over dosage timing [8]. Thanks to advancements in polymer science and nanotechnology, researchers have been able to develop “smart” hydrogels, endowed with tunable and “on-demand” drug release patterns [3,4,7,9,10]. These smart gels, also referred to as stimuli-responsive hydrogels, are engineered to respond to various stimuli such as temperature, pH, electromagnetic radiation, magnetic field, or the presence of specific biological factors. The fundamental aspect of smart hydrogels is their ability to modify their mechanical properties, swelling ability, hydrophilicity, or bioactive molecule permeability, influenced by various stimuli. This property enables them to be triggered to release drugs in a controlled and targeted manner, thus enhancing the precision and effectiveness of drug delivery.

Smart hydrogels exhibit versatile applications, spanning tissue engineering, cell cultures, and innovative drug delivery systems (DDSs) [3,11,12,13]. By responding to physical, chemical, and biological stimuli, these hydrogels can effectively release active substances, making them ideal for disease treatment. Furthermore, their mechanical properties are comparable to those of biological tissues, making them well-suited for mimicking natural living tissues [6].

In DDSs, smart hydrogels deliver substantial advantages [3,14,15]. They respond to stimuli such as pH, temperature, light, redox, and biomolecules, leading to improved drug efficacy and reduced side effects. Moreover, they enable precise drug release over time, reducing the need for frequent dosing. This highlights the immense potential of smart hydrogels in revolutionizing targeted drug delivery by improving efficacy, minimizing side effects, and enabling personalized treatments.

Recent advancements in the development of materials capable of responding to specific stimuli have paved the way for innovative smart hydrogels and nanogels that exhibit sensitivity to particular signals and pathological conditions [16,17]. The aim of this review is to highlight the “smart” stimuli-responsive mechanisms underlying the design of intelligent hydrogels and explore their current applications in treating various diseases, along with the current challenges and future perspectives in this field. While many stimuli can trigger responses in smart hydrogels, our focus in this review is on the most commonly employed stimuli, including pH, temperature, light irradiation, redox conditions, and biomolecules in drug delivery systems.

## 2. Hydrogel Systems Used for Drug Delivery

### 2.1. Definition and Classification of Hydrogel and Nanogel Systems

**Figure 1:**
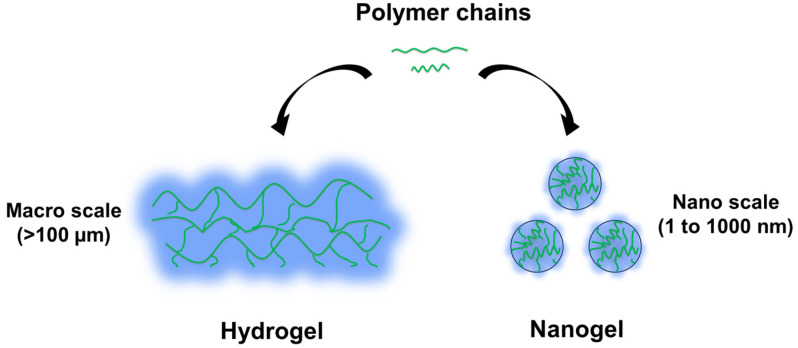
Representative scheme of hydrogel and nanogel. They share the same hydrophilic 3D network structure but differ in scale.

As shown in Figure 1, both hydrogels and nanogels are hydrophilic polymeric networks, differing primarily in scale [6,18,19,20,21,22]. Hydrogels are three-dimensional networks with remarkable water absorption, swelling capacity, and biocompatibility. In contrast, nanogels are a distinct subset, ranging from 1 to 1000 nm in size, falling within the nanometer to sub-micrometer range. Both hydrogels and nanogels possess a 3D structure with crosslinked amphiphilic or hydrophilic polymer chains, enabling the encapsulation of both hydrophilic and hydrophobic drugs. They can also be engineered to be responsive to specific stimuli and can be modified with ligands for active targeting [23]. Nanogels exhibit a specific surface area and inner space, increasing the stability of loaded drugs and enabling controlled or sustained drug release. In summary, nanogels are hydrogel nanoparticles that are considered the next generation of DDSs, due to their superior properties, including high drug loading capacity, low toxicity, and stimuli responsiveness.

### 2.2. Unique Properties of Nanogels Imparted by Size Reduction to the Nanoscale

Despite the many similarities between hydrogels and nanogels, the latter possess some unique properties that stem from their combination of nano-size and hydrogel characteristics, rendering them highly promising candidates for DDSs [24,25,26]. The reduction in hydrogel particle size to the nanoscale imparts several unique properties to nanogels, including precise control over their interfacial properties, improved mechanical strength, and enhanced responsiveness to external stimuli. Specifically, these properties encompass: (1) an increased surface-area-to-volume ratio, which enhances the drug loading amount and release efficiency [27]; (2) controlled interfacial properties and the ability to modify or functionalize particle interfaces [28,29]; (3) a suitable nanoscale size, which enables nanogels to penetrate biological barriers and reach specific target sites in the body, such as tumors, facilitating cell uptake and tumor penetration [30]; (4) stimuli-responsive behaviors that can be controlled by selecting constituent polymer and crosslinker components, allowing for a desired response at the site of action, and enabling nanogels to actively participate in the intended function of the carrier system, rather than being passive carriers of their cargo [31]; (5) improved stability of drugs and increased circulation time in the bloodstream due to their small size, which can improve drug efficacy [32]; and (6) enhanced biocompatibility and biodegradability due to the small size, which reduces toxicity [33].

## 3. Stimuli-Responsive Hydrogels in Drug Delivery

**Figure 2:**
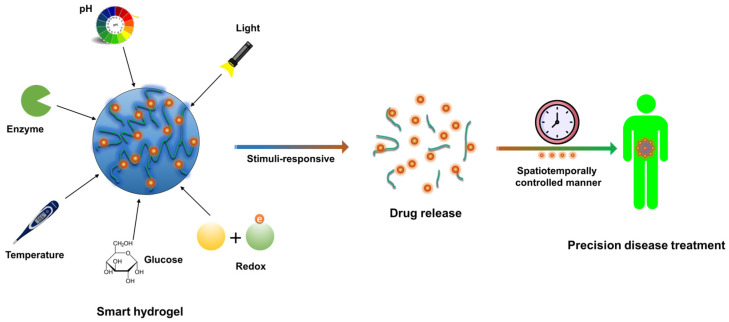
An overview of stimuli-responsive hydrogels/nanogels. The encapsulated drug can be triggered to release in a spatiotemporally controlled manner and delivered to the target site to achieve precise disease treatment.

Stimuli-responsive hydrogels, also known as smart hydrogels, exhibit responsiveness to diverse external stimuli [2,7,34,35,36,37]. These gels can undergo reversible or irreversible changes in physical or chemical properties upon exposure to stimuli, enabling a highly controllable drug release pattern. This capability contributes to achieving precise drug administration and enhancing treatment effectiveness and safety, as depicted in Figure 2. This review extensively examines the key principles governing responsive hydrogel design, focusing on prominent stimuli such as pH, temperature, light irradiation, redox conditions, and specific biomolecules (as shown in Table 1).

### 3.1. pH-Sensitive Hydrogels

Biological fluid pH is a significant chemical property that holds immense potential in the drug delivery field. pH-responsive hydrogel and nanogels have been developed as intelligent drug delivery carriers, capable of exhibiting swelling or shrinking behavior in response to changes in pH [71,72,73,74]. Thus, these hydrogels are able to release encapsulated drugs in a site-specific manner.

The pH-responsive mechanisms can be divided into two types: First is employing polymers with ionizable moieties like amines and carboxylic acids, protonated or deprotonated at various pH values [38,39]. For instance, when the pH level is below the pKa of the basic functional groups, such as poly[(2-dimethylamino)ethyl methacrylate] (PDMA), the hydrogel becomes protonated and forms a positively charged polymer chain, leading to swelling due to electrostatic repulsion between the charged groups. Conversely, when the pH level is above the pKa of the functional groups in the hydrogel, the hydrogel is deprotonated and shrinks due to electrostatic attraction between the charged groups. Polyacids, such as poly(methacrylic acid) (PMAAc), behave inversely, accepting protons at low pH and releasing protons at neutral and high pH. Therefore, pH shifts can alter the interaction of hydrogel polymer chains, triggering drug release from pH-sensitive hydrogels. However, it is important to note that the response of ionizable moieties to pH can be influenced by other factors, such as temperature and ionic strength [40], making it challenging to control drug release in complex conditions. Second is utilizing polymers that contain acid-labile linkages [41,42]. These covalent linkages are stable at physiological pH but cleave as pH decreases, leading to polymer chain degradation or aggregate dissociation. Various chemical moieties, including anhydrides (DMMA, succinic anhydride, cis-aconitic anhydride, and cis-cyclohexene-1,2-dicarboxylic anhydride) [43,44], hydrazone [45,46], and imine [47], can be adopted to create acid-labile linkages. Compared to the first approach, this approach is more flexible, as it allows for the selection of different acid-labile linkages conjugated with various polymers. As a result, it offers more precise control of drug release in response to the acidic environment in pathological conditions [41,71]. However, note that acid-labile linkages can be unstable, potentially releasing drugs before reaching the acidic target sites. For example, Zou et al. reported that the drug conjugate poly(ethylene oxide)-block-polyphosphoester-graft-PTX (PEO-b-PPE-g-PTX G2) with acid-labile linkages exhibited 20% PTX release in 8 days under neutral conditions, even though it showed accelerated drug release under acidic conditions (approximately 50% PTX release in 8 days under acidic conditions) [48].

It is noteworthy to mention that the conventional polymerization methods for acid-labile linkages may raise toxicity issues [73]. Over the past few years, one of the main advancements in this field has been the development of pH-responsive hydrogels and nanogels with improved biocompatibility and drug-loading capacity. Researchers have explored natural polymers like chitosan and alginate, along with biocompatible polymers such as hydroxypropyl methylcellulose (HPMC), to create pH-sensitive hydrogels that are both biodegradable and safe [75,76,77].

### 3.2. Thermoresponsive Hydrogels

Due to their inherent physiological condition and convenient administration, temperature or thermoresponsive hydrogels have been widely utilized in the field of drug delivery. These hydrogels can undergo phase transitions from a swollen state to a collapsed or shrinking state, or vice versa, in response to changes in temperature. Using monomers such as N-isopropylacrylamide (NIPAM) and cross-linkers such as methylene bisacrylamide (MBA) or poly(ethylene glycol) diacrylate (PEGDA), temperature-sensitive hydrogels are commonly synthesized through free radical polymerization [49,50].

Sol–gel phase transitions in these hydrogels are driven by changes in the interaction between their hydrophobic and hydrophilic segments with water molecules, leading to changes in the solubility of the cross-linked network and resulting in sol–gel phase transition [78]. The sol phase is a flowing fluid, while the gel phase is non-flowing and maintains its integrity. Hydrogels can form either above the lower critical solution temperature (LCST) or below the upper critical solution temperature (UCST), depending on the specific composition and ratio of hydrophilic and hydrophobic components. The polymer is soluble below the LCST, but as the temperature rises above the LCST, the hydrogel begins to shrink, becoming hydrophobic and insoluble, resulting in the formation of a gel [79]. Conversely, cooling the polymer solution below the UCST triggers the formation of a hydrogel. Near the critical temperature, the polymer undergoes a phase change from a soluble state (random coil) to an insoluble state (collapse or micelle form) [80]. The phase transition temperature (PTT) of a temperature-sensitive hydrogel can be adjusted by changing the chemical composition of the polymers, the concentration of anionic monomer, or the ratio of hydrophilic/hydrophobic groups in the gel materials [81].

While natural thermoresponsive polymers, such as some polysaccharides (e.g., agarose, amylose, amylopectin, and some cellulose derivatives) and certain proteins (e.g., gelatin, collagen, and elastin-like polypeptides), can form thermo-reversible hydrogels, they generally exhibit weak mechanical strength and slow temperature responses, necessitating chemical modification to improve their properties [51,82]. In contrast, synthesized polymers, such as poly(N-isopropylacrylamide), poloxamers, and PLGA-PEG-PLGA triblock polymers, offer greater adjustability in physical properties. They can be incorporated into natural or synthetic polymers to introduce thermoresponsive qualities, enabling controlled drug release. For example, N-isopropylacrylamide (NIPAM) can be grafted with polymers like alginate [83], chitosan [84], hyaluronic acid [85], or PEG [50] to impart thermoresponsive properties to these polymers.

Regarding biocompatibility, it is widely acknowledged that the NIPAM monomer possesses toxicity. However, the PNIPAAm polymers with high molecular weight or the grafted pPNIPAAm with other polymers have exhibited notable biocompatibility in several studies [86,87,88]. Examining the cytocompatibility and hemocompatibility of thermoresponsive PNIPAAm and PNIPAAm-PLLA-PNIPAAm triblock copolymers, Su et al. discovered that the latter demonstrated exceptional biocompatibility, thus offering potential for targeted drug delivery [86]. In a separate study, Yogev et al. investigated the biocompatibility of thermoresponsive polymers, including PNIPAAm, poly(ethylene glycol)-poly(propylene glycol)-poly(ethylene glycol) triblock copolymer, poly(lactic acid-co-glycolic acid), and poly(ethylene glycol) triblock copolymer, both in vitro and in vivo. They concluded that all tested materials demonstrated satisfactory biocompatibility in vivo [86]. The toxicity of commercially available PNIPAAm probably results from the release of NIPAM monomer and impurities in the pNIPAAm.

### 3.3. Photo-Responsive Hydrogels

Light exposure serves as a stimulus for drug release from hydrogels, encompassing UV, visible, and infrared light [52,53,54,55]. As a non-invasive and efficient external trigger, light optimally controls drug release from hydrogels, enhancing therapeutic effectiveness and minimizing side effects by regulating drug distribution within the body. Photo-responsive hydrogels undergo phase transition, stiffness alteration, or biochemical activation upon light exposure, prompting drug release exclusively in illuminated areas. This method offers accurate and non-contact drug delivery, applicable in a range of medical scenarios such as chemotherapy, immunotherapy, photodynamic therapy, gene therapy, and wound healing [89,90,91,92].

Various photosensitive chemical moieties can be used for photo-responsive functionality, including o-nitrobenzyl ester linkers [56], arylazopyrazole [57], azobenzene [58], and photocleavable proteins such as PhoCl [59]. These chemical moieties allow hydrogels to respond to either UV light or visible light, leading to reversible changes in their properties. Photo-responsive hydrogels are fabricated by integrating these photosensitive components into their polymeric structures through diverse approaches. These approaches can be categorized into three groups based on their mechanisms: photoisomerization, photochemical reactions, and photothermal reactions [55]. Photoisomerization is a process in which a molecule undergoes a structural transformation upon light absorption. Upon exposure to light, the photosensitive components within the hydrogel experience photoisomerization, which induces a change in the hydrogel’s structure and initiates drug release [53]. Photochemical reactions, on the other hand, refer to chemical reactions initiated by light absorption. In light-responsive hydrogels, photosensitive moieties undergo photochemical reactions upon exposure to light. This results in the cleavage of chemical bonds, leading to drug release [93]. Photothermal reactions involve the absorption of light by a material, causing an increase in temperature. In light-responsive hydrogels, this resulting temperature rise brings a change in the hydrogel’s structure and then triggers drug release [3]. It should be noted that photo-responsive processes may be reversible or irreversible. Reversible photo-responsive hydrogels can undergo a gel-to-sol transition or sol-to-gel transition upon light exposure. This can be used to accelerate drug release and achieve a step-by-step release pattern. Irreversible photo-responsive hydrogels undergo a permanent change upon light exposure, which can be used for one-time drug release or gradual release through hydrogel degradation.

Photo-responsive hydrogels can respond to varying light wavelengths, including UV, visible, and near-infrared (NIR) light, depending on the adopted photosensitizers. UV light provides greater energy for photo curing compared to the other two longer-wavelength lights, resulting in a higher curing speed and efficiency [94]. However, UV light is associated with DNA, tissue damage, and limited tissue penetration due to light absorption and scattering by water and other substances [95]. UV light photocuring can be used when enhanced photochemical reactions are required, such as in vivo 3D printing of bone substitutes through photo-fabrication technologies [94]. On the other hand, near-infrared (NIR) light within the 700 to 1000 nm range is more appealing for DDSs compared to other wavelength spectra. It does not cause harm to living cells or tissues, and it also possesses superior tissue penetration capability [96]. As a result, an increasing number of NIR-sensitive photo-responsive hydrogels are being developed.

Recent advances in material science have led to diverse new photosensitizers for NIR-sensitive photo-responsive hydrogel preparation, such as rare metal nanostructures and black phosphorus nanoparticles [52,54]. Qiu et al. have developed an innovative photothermal hydrogel by integrating black phosphorus into hydrogel nanostructures [54]. The hydrogel can be activated by NIR light with a wavelength of 808 nm, which causes the drug-loaded hydrogel nanostructures to soften and melt, ultimately resulting in drug release. Qiu used a power density of 1 W/cm^2^, while Auge et al. designed a more efficient photothermal hydrogel, notably lowering the power density to 0.16 W/cm^2^. They formulated a nickel-bis(dithiolene) complex that can undergo a volume phase transition and release loaded hydrophobic dye molecules upon NIR light exposure. They also extended the working NIR spectral region to 1000 nm [52].

### 3.4. Redox-Responsive Hydrogel

Innovative DDSs have emerged with the development of redox-responsive hydrogels [60,97,98]. These hydrogels, when subjected to specific biological redox stimuli, can rapidly release encapsulated drugs at the target site [62]. The redox-responsive behavior of the hydrogel/nanogels is achieved through the incorporation of specific chemical moieties. One such chemical moiety is the disulfide linker, which can be cleaved in the presence of reducing agents such as glutathione (GSH) [60,61]. Another widely used chemical moiety is the selenide group, which can be responsive to reactive oxygen species (ROS) such as hydrogen peroxide (H_2_O_2_) [63].

The redox-responsive hydrogel carriers can be specifically designed for specific purposes. For gene drug delivery, Zhao et al. devised gelatin/silica-aptamer nanogels that can selectively release siRNA into the cytosol in nucleolin-positive cells (A549) triggered by GSH [60]. To encapsulate therapeutic proteins, Schotz et al. crafted polyglycerol-based redox-responsive nanogels using inverse nanoprecipitation and inverse electron-demand Diels–Alder cyclizations between methyl tetrazines and norbornene. The encapsulated cytochrome C was released at the action site under physiological reductive conditions [64]. For intracellular delivery of cationic drugs, Maciel et al. synthesized redox-sensitive nanogels (AG/Cys) through in situ cross-linking of alginate using cystamine as a cross-linker via a mini-emulsion method. The cationic doxorubicin was encapsulated via electrostatic interactions, and the encapsulation efficiency was up to 95.2 ± 4.7% [55,56]. Natural polymer-based materials, such as hydroxypropyl cellulose (HPC)-based grafted copolymers, were also adopted for biodegradable and biocompatible nanogels [97,99]. These advancements in redox-responsive hydrogels/nanogels hold more promise for effective and targeted DDSs.

### 3.5. Biomolecule-Responsive Hydrogels

#### 3.5.1. Enzyme-Responsive Hydrogels

Enzyme-responsive hydrogels have been developed to respond to enzymatic activity in specific environments, enabling the release of encapsulated therapeutics [65,66]. These hydrogels offer a distinct advantage due to their reliance on endogenous enzyme expression. One mechanism involves designing the hydrogel matrix to degrade under specific enzymes, allowing for controlled therapeutic release. Yang et al. devised enzyme-responsive nanogels (EPNGs) cross-linked with cinnamyloxy groups in PEGylated hyaluronic acid, which are sensitive to hyaluronidase [66]. These EPNGs exhibit high loading efficiency and excellent stability in various biological media. However, they degrade rapidly within tumor cells that overexpress hyaluronidase, allowing for rapid release of encapsulated cytochrome C. Another mechanism involves covalently linking therapeutics to the hydrogel scaffold with enzymatically sensitive cross linkages. Amer et al. created a PEG hydrogel delivering the anticancer drug doxorubicin, which was covalently attached to the hydrogel via the MMP-sensitive peptide linker, C-VPLS↓LYSG-C [67]. The hydrogel was able to release doxorubicin in the presence of MMP-2 and MMP-9, significantly reducing tumor growth in a breast cancer mouse model [57].

#### 3.5.2. Glucose-Responsive Hydrogels

In response to real-time blood-glucose levels, glucose-responsive carriers have been developed to facilitate insulin release [68,100,101]. These carriers can be designed using motifs such as glucose oxidase (GOx), phenylboronic acid (PBA), or concanavalin A (Con A) [102], which can detect glucose levels in their surroundings. By integrating these motifs into the hydrogel matrix, they can be utilized to trigger insulin release in a glucose-responsive manner, leading to improved control of blood-glucose levels and reduced risk of hypoglycemia [103].

The GOx enzyme catalyzes the conversion of glucose to gluconic acid in the presence of oxygen, causing local physiochemical changes such as pH, H_2_O_2_ levels, or alterations in oxygen concentrations. These changes are then harnessed to induce insulin release in a glucose-responsive fashion. It should be noted that increasing levels of H_2_O_2_ and gluconic acid can hinder GOx activity, thereby diminishing the hydrogel’s property changes and reducing sensitivity to glucose. To address this issue, Gordijo introduced the enzyme catalase (CAT) to convert H_2_O_2_ to water and O_2_, enhancing GOx activity [104]. Based on this, Gu et al. engineered glucose-responsive closed-loop insulin microgels (256 ± 18 μm) containing a pH-responsive chitosan hydrogel and GOx enzyme nanocapsules through a one-step electrospray process [69]. They observed a decrease in blood-glucose levels in a type 1 diabetes mouse model. Moreover, improving the stability of the GOx enzyme, which is inherently unstable, can be achieved by using high hydrostatic pressure and hydrophobic modification [105]. Kim et al. developed glucose-responsive hydrogels cross-linked by citric acid, embedding GOx within the hydrophobic β-CD cavity to enhance its stability and achieve long-term glucose monitoring [100].

To overcome the instability of GOx enzyme, a synthetic glucose-responsive motif, phenylboronic acid (PBA), was adopted. As a derivative of boronic acid, PBA can form reversible covalent bonds with diols like glucose. When glucose levels are high, competition for saccharide binding sites on glucose-binding molecule-polymer complexes lead to separation of the complex, triggering insulin release. Wang et al. introduced the hydrophilic monomer N-vinyl-2-pyrrolidone and the amino-containing monomer N,N-dimethylaminopropyl acrylamide to PBA-based polymers to create glucose-sensitive microgels that respond to physiological temperature and pH [68]. These microgels, formed by a reversed-phase microemulsion method, create a dense network at low glucose levels that can encapsulate insulin and prevent leaks but disrupt the bond at high glucose levels, thus rapidly releasing insulin. It should be noted that PBA lacks specificity for glucose, despite being more stable than the GOx enzyme.

Similarly, concanavalin A (Con A), a natural carbohydrate-binding protein, can reversibly attach to glucose and other saccharides. Con A forms a tetrameric structure and can bind to four glucose molecules, acting as a macromolecular crosslinker. When Con A is integrated into the hydrogel matrix, it forms a glucose-binding element that can release insulin when glucose levels change. Variations in the ratio of uncharged and charged borates, influenced by glucose, impact polymer solubility and facilitate glucose-responsive insulin release. Based on Con A’s glucose-responsive property, Lin et al. developed a pullulan-based glucose-responsive hydrogel by covalently modifying Con A with a pullulan derivative containing COOH groups, allowing for intelligent, controlled insulin release upon glucose concentration shifts [70]. The hydrogel swells and releases insulin when glucose levels are high, but when glucose levels are low, insulin release is reduced.

These discoveries contribute to the progress of hydrogel systems that can adapt to glucose changes and autonomously regulate the release of anti-diabetic medications according to blood glucose levels [106]. These glucose-responsive hydrogels hold potential for diabetes management, potentially enhancing patient outcomes.

### 3.6. Multi-Responsive Hydrogels

The multi-responsive hydrogel/nanogel has been proposed to enable a response to different triggers such as temperature, light, redox conditions, etc. [107,108]. This is crucial for achieving precise control over drug release. In a study by Gao et al., a random copolypeptide was designed through ring-opening copolymerization, incorporating poly(methoxy-diethylene glycol–L-glutamate)-co-poly(S-(o-nitrobenzyl)-L-cysteine). This copolypeptide displayed quadruple thermo–photo–redox-responsive self-assemble behavior, forming nanogels in water [109]. These nanogels demonstrated excellent biocompatibility and degradability, making them promising for DDSs and tissue engineering scaffolds. Another example by Jo et al. involved the development of a smart hydrogel responsive to multiple stimuli, including pH, reducing agents, oxidizing agents, and NIR irradiation. The hydrogels showed a rapid release of doxorubicin (DOX) in acidic conditions (pH 5), with reducing agents (10 mmol DTT), in oxidizing medium (0.5% H_2_O_2_), as well as upon NIR irradiation [110]. These multiple controlled-release mechanisms enhance targeted drug delivery and help mitigate potential side effects.

## 4. Applications in Treating Different Diseases

Comprehending the unique biological or pathological conditions of specific diseases is a fundamental requirement for the creation and application of smart hydrogels in drug delivery and therapy [35,111]. With increasing insights into the distinct characteristics of each disease’s microenvironment, researchers can tailor smart hydrogels with appropriate functional groups that can react to particular triggers like pH, temperature, redox reactions, enzymes, or disease-specific signals. We discussed the unique pathological or biological conditions of each disease and how to use them to design corresponding smart hydrogels, summarizing the findings in Table 2. This customization of hydrogels to match individual disease contexts can result in improved therapeutic outcomes, reduced side effects, and better treatment compliance. This advancement ultimately could revolutionize the DDSs and disease management.

### 4.1. Oral Disease

The physiological actions within the oral cavity, such as saliva production and chewing, play a substantial role in minimizing the stay of therapeutic medications in periodontal disease. This has resulted in significant interest in utilizing hydrogels for oral disease treatment due to their excellent bioadhesion, biocompatibility, and ease of administration. Smart hydrogels have been researched for addressing oral diseases such as periodontitis and implantitis [7]. These responsive hydrogels can undergo reversible sol–gel transitions in situ and control drug release upon exposure to varied triggers such as temperature, pH, ROS, or enzymes, which can significantly improve the efficacy of oral disease treatment [134].

Matrix metalloproteinase-8 (MMP-8) acts as a key collagenase and serves as an indicator of inflammation and prevention in periodontal and peri-implant diseases. Guo et al. designed an MMP-8-responsive hydrogel for on-demand intraoral drug release. This hydrogel was designed to be cleaved by MMP-8, allowing for adaptive degradation in response to chronic periodontitis and peri-implantitis [112]. The release of drugs could be adjusted by modifying the loading technique and MMP-8 concentration.

Furthermore, oral inflammation can lead to pH changes in the microenvironment of periodontitis sites [113,135]. The pH change within the periodontitis area is linked to the severity of the disease [113]. For instance, patients with generalized chronic gingivitis were found to have a more alkaline pH compared to the control group, while those with generalized chronic periodontitis displayed a more acidic pH. Therefore, a pH-sensitive hydrogel that can encapsulate therapeutic drugs and enable gel formation in situ could be a suitable approach for treating periodontitis. Chang et al. developed a pH and temperature-responsive injectable hydrogel for periodontitis treatment [114]. The hydrogel maintained its fluidic state at low temperatures but rapidly transformed into a gel at 37°C, leading to a faster release of the encapsulated drug (naringin) at pH 5.5 to 6.5 in the inflamed site. An in vivo study indicated that the smart hydrogel reduced bone loss and inflammation associated with periodontitis. These studies underscore the potential of stimuli-responsive hydrogels for precise and controlled drug delivery in addressing oral diseases.

### 4.2. Cancer

In spite of the availability of diverse treatments, achieving optimal efficacy in anti-cancer therapies remains challenging [136]. The primary hurdles in effective cancer treatment involve the emergence of resistance in cancer cells to chemotherapy, compromised intracellular drug transport, deactivation of therapeutic agents, and pronounced systemic and organ toxicity [137,138]. As a solution, hydrogels/nanogels have been harnessed as drug carriers, offering controlled drug release at tumor sites with reduced toxicity and a response to specific triggers, making them more suitable for cancer treatment compared to other carriers. These smart nanogels can respond to stimuli within the tumor microenvironment, such as changes in pH, temperature, light, redox, etc., enabling a precisely managed drug release for cancer therapy [2,13,26,36,139].

To start, tumor cells often exhibit imbalanced enzyme levels that considerably differ from normal cells, disturbing cellular equilibrium [140]. Tumor cells actively release matrix metalloproteinases (MMPs) and other proteolytic enzymes into the extracellular matrix, causing its disintegration and creating space for tumor growth [141]. The dysregulated MMP level can be exploited to design MMP-sensitive hydrogels, typically formulated by cross-linking polymer chains with specific peptide-bound amino acid fragments sensitive to MMP activity [142]. For example, Li et al. developed an MMP-2 responsive hydrogel composed of hyaluronic acid (HA) and an MMP-2-sensitive peptide. This hydrogel displayed a responsive drug release pattern in vitro [115]. In vivo studies demonstrated faster hydrogel degradation at the tumor site, resulting in boosted drug release and tumor growth suppression without damaging the organs.

Moreover, tumor tissues generally possess a lower pH compared to normal tissues [143]. This pH discrepancy can be exploited to devise pH-sensitive hydrogels that modulate the delivery and release of anti-tumor drugs specifically at tumor sites. Liu designed a pH-responsive peptide nanogel to concurrently release two anti-tumor drugs, gemcitabine and paclitaxel, at the same location to enhance the anti-tumor effect and prevent drug resistance [116]. In vivo experimentation confirmed the nanogel’s ability to reach the tumor site, ensuring gradual and continuous release of the two drugs in the tumor microenvironment.

Furthermore, a majority of tumors exhibit elevated GSH levels, which contribute substantially to tumorigenesis and inflammation responses within the tumor microenvironment [144]. As the most prevalent non-protein thiol, GSH governs cellular redox equilibrium and protects cells from harm induced by lipid peroxides, reactive oxygen, and nitrogen species. Notably, a GSH-responsive nanogel was conceived by Tian et al., constructed from poly(ethylene glycol), diglycidyl ether, and cystamine double-crosslinked hyaluronic acid (HA) [117]. The nanogel specifically targets tumors via HA-receptor-mediated endocytosis and exhibits responsive swelling upon cleavage of disulfide bonds when confronted with high GSH concentrations in tumor cells. This process triggers rapid drug release, resulting in greater cytotoxicity against tumor cells compared to normal cells. The utilization of redox-responsive nanogels also offers a platform to combat drug resistance, as elevated GSH levels in cancer cells amplify drug release and elevate cytotoxicity against tumors [98].

Lastly, solid tumors often suffer from insufficient growth of new blood vessels, leading to inadequate oxygen supply and a hypoxic environment [145]. Hypoxia fosters tumor progression, metastasis, and resistance to chemotherapy and radiation therapy. Specifically, azobenzene (Azo) polymers adopting a donor/acceptor substitution pattern have demonstrated remarkable efficacy in enzymatically triggered Azo cleavage reactions under hypoxic conditions [146]. Therefore, a hypoxia-sensitive hydrogel based on azobenzene could be engineered to precisely release drugs upon sensing low oxygen levels in target sites [118,119,120]. In line with this, Si et al. introduced a hypoxia-sensitive nanogel system through host–guest interactions between Azo and β-cyclodextrin (βCD), coupled with poly (L-glutamic acid)-graft-poly (ethylene glycol) methyl ether (PLG-g-mPEG) [121]. The cross-linkage between Azo and βCD is disrupted in the presence of nitroreductase (NTR), an enzyme excessively expressed in hypoxic tumors, thereby releasing the encapsulated ribonuclease A (RNase) as a treatment for breast cancer. The in vivo investigation demonstrated the potential of the hypoxia-sensitive supramolecular nanogel in delivering RNase as a breast cancer therapy, noting a tumor suppression rate of 68.7% in the nano-RNase treated group, whereas free RNase treatment failed to inhibit tumor growth.

### 4.3. Wound Healing and Topical Application

The wound healing process can be categorized into acute and chronic healing. Acute wounds, resulting from skin breakage or puncture, typically heal quickly and are categorized based on their causes, such as surgical incisions, thermal injuries, abrasions, lacerations, and gunshot wounds [147]. In contrast, chronic wounds, often associated with conditions like diabetes and obesity, require a longer healing duration due to the disruption of the normal healing cascade caused by extensive inflammation, impaired angiogenesis, etc. [148,149]. Wound healing involves various cell types and distinct phases—hemostasis, inflammation, proliferation, and maturation [150]. Traditional wound dressings like bandages, dumb hydrogels, and foams are insufficient in addressing the wound healing process [151]. Smart hydrogels have emerged as wound dressings that can interact with wounds, detecting and responding to changes in the wound condition, facilitating effective healing [152].

For example, chronic wounds, like diabetic foot ulcers, often lack oxygen, hampering healing [153]. Xiong et al. have developed a smart hydrogel that converts excess hydrogen peroxide into oxygen, mitigating harmful effects and providing oxygen for wound healing [122]. The designed HA@MnO_2_/FGF-2/Exos hydrogel dressing, synthesized via Schiff base reaction, effectively enhances diabetic wound healing by providing antibacterial action, catalyzing H_2_O_2_ to O_2_ conversion, and releasing substances that boost angiogenesis and speed up epithelization. In vitro and in vivo data demonstrate the hydrogel’s biocompatibility with potent early-stage anti-infection, anti-oxidation, and anti-inflammation effects, further advancing angiogenesis and wound repair.

Moreover, wound tissues display distinct pH and reactive oxygen species (ROS) levels from healthy tissues. In chronic ulcers, the wound fluid’s redox environment exhibits increased radical scavenging activity and glutathione levels, indicating compensatory mechanisms against inflammation [154]. Additionally, the wound surface exhibits a decreased pH value, and this pH has been found to be correlated with healing time. A lower pH value is associated with faster healing [155,156]. Therefore, smart hydrogels incorporating pH and ROS responsiveness, along with drugs having anti-inflammatory and antioxidant functions, were developed. Wu et al. engineered a pH/ROS dual-responsive injectable glycopeptide hydrogel based on phenylboronic acid-grafted oxidized dextran and caffeic acid-grafted e-polylysine, demonstrating inherent antibacterial and antioxidant capabilities [123]. Similarly, Li et al. developed a self-healing, injectable, and pH-responsive hydrogel for treating diabetic foot ulcers (DFUs). This pH-responsive hydrogel was created through the interaction of N-carboxyethyl chitosan, hyaluronic acid-aldehyde (HA-ALD), and adipic acid dihydrazide (ADH), forming reversible dynamic bonds like acylhydrazone and imine bonds [72]. These responsive hydrogels have shown promise in expediting the wound healing process, especially in managing chronic wounds.

Furthermore, the concept of self-healing hydrogels has emerged, allowing them to autonomously repair themselves when damaged, thus enhancing their stability and resilience when promoting chronic wound healing [157,158,159]. The use of composite materials has also been explored to enhance the mechanical properties of these hydrogels [160,161]. This self-healing mechanism relies on both covalent and non-covalent interactions, enabling the hydrogel to regain its original mechanical attributes, including shape, injectability, and stretchability. This quality makes them superior in terms of durability and steadfastness.

### 4.4. Neurological Disorders

Drug resistance in neurological diseases is a significant challenge that occurs at various levels, including genomic and proteomic levels, affecting cellular transporters and disrupting signaling pathways [162,163]. This resistance hinders the therapeutic effect of drugs and leads to severe health complications. Stimuli-responsive hydrogels exhibit the potential to mitigate drug resistance in neurological disorders by orchestrating controlled drug release and targeted delivery. These hydrogels can be fine-tuned to respond to distinct stimuli, like pH, temperature, or enzymes, ensuring precise drug release at designated sites [124,164]. Another challenge in neurological disease treatment is the formidable blood–brain barrier, which shields the brain but also prevents many drugs from entering the brain [165]. Hydrogels offer distinct advantages for treating neurological conditions, primarily due to their capability to deliver bioactive agents and cells across the blood–brain barrier [18]. Consequently, hydrogels are emerging as promising candidates to address prevalent neurological diseases—ranging from Alzheimer’s, Parkinson’s, spinal cord injuries, and stroke to brain tumors [166]. Furthermore, hydrogels can mimic the properties of the central nervous system’s extracellular matrix, making them ideal carriers for drug delivery and tissue regeneration. Specifically, injectable hydrogels can minimize invasiveness during administration and can encapsulate exogenous cells and therapeutic molecules, while providing a permissive environment for cell survival and propagation [167].

Engineered stimuli-responsive hydrogels show promise for restoring spinal cord injuries (SCIs). They can bridge spinal cord lesions, mimicking the mechanical and electrical properties of native spinal cords. Moreover, these hydrogels facilitate direct drug release within the injury microenvironment based on SCI activity. Notably, Fan et al. innovated an MMP-responsive bionic hydrogel with favorable mechanical and conductive properties akin to native spinal cords, enabling on-demand release of biological agents (GST-TIMP-βFGF) in response to the SCI microenvironment [124]. The in vivo study conducted on SCI model rats exhibited that the smart hydrogel could inhibit MMP levels, promote axon regrowth and angiogenesis, and enhance locomotion recovery post SCI.

In a broader perspective, smart hydrogel composites can be engineered for precise and long-term delivery of bioactive substances through multi-stimulus responsiveness. Michael C. Koetting et al. synthesized a nanogel through the radical polymerization of poly(N-isopropylacrylamide), which was then incorporated into a hydrogel composed of poly(acrylic acid) grafted onto κ-carrageenan polysaccharide, using magnetic iron oxide nanoparticles as crosslinkers [125]. This system leverages responsive functional polymers to attain pH, thermal, and magnetic sensitivity, ensuring over 11 days of controlled drug (levodopa) release for neurological disorder treatment.

### 4.5. Diabetes

Diabetes represents a substantial contemporary healthcare challenge, characterized by disrupted glucose metabolism leading to conditions such as hyperglycemia, glycosuria, and hyperlipidemia [168]. The global annual cost for treating diabetes reaches billions of dollars. While multiple daily insulin injections are commonly used, they are invasive and can lead to suboptimal patient compliance [169]. Insulin delivery has evolved from direct injections to advanced hydrogel-based methods that respond to various stimuli, including glucose, pH, electric, or magnetic fields, triggering insulin release. For instance, glucose-sensitive hydrogels can mimic the behavior of pancreatic beta cells by releasing insulin upon changes in glucose levels [126]. Similarly, pH-sensitive polymeric hydrogels can facilitate oral insulin delivery, shielding insulin from stomach acid degradation and enabling its release in the neutral pH of the intestine [74]. Among these approaches, glucose-responsive hydrogels and artificial beta cell therapy stand out as the most promising solutions.

Glucose-responsive hydrogels with a closed-loop system integrate continuous glucose monitoring with automated insulin delivery, creating a feedback loop that mimics the pancreas’ natural regulation [101]. Insulin is released in response to real-time changes in glucose levels, offering self-regulated insulin delivery, and providing potential for effective glycemic control with minimal patient discomfort [5,170]. Furthermore, these smart hydrogels can be adapted to suit various medical scenarios, such as microneedle patches, injectable micro/nanohydrogels, oral/nasal/pulmonary microcapsule formulations, and in vivo implants [74,126,127,128,129,130,131,132].

For insulin-producing cell therapies, hydrogels can be used to encapsulate insulin-secreting cells and protect them from the immune system [171,172,173]. They offer a natural microenvironment akin to the cells’ native extracellular matrix (ECM), supporting long-term cell implantation within the challenging in vivo conditions. These hydrogels can be designed to facilitate nutrient and oxygen transfer to the encapsulated cells while mitigating foreign body responses. Various biocompatible hydrogel materials, including alginate, chitosan, and polyethylene glycol (PEG), have been explored in these systems. Although there are still obstacles to overcome before these carriers can be used in clinical applications, optimizing delivery systems is anticipated to have a considerable impact on the treatment of type 1 diabetes.

### 4.6. Cardiovascular and Cerebrovascular Diseases

Cardiovascular diseases (CVDs), including atherosclerosis, vascular inflammation, and rheumatic heart disease, have stood as the foremost global cause of mortality for many years [174]. While stem cell transplantation and growth factor therapy hold promise in treating these diseases, their efficacy is impeded by the low survival rates of cells/growth factors at injury sites. To address this challenge, the emergence of smart hydrogels has opened new avenues for CVD treatment [133,175,176]. Stimuli-responsive hydrogels offer a smart solution, enabling precise control over the spatiotemporal release of therapeutic agents, a capability absent in traditional hydrogels.

For instance, myocardial infarction (MI) can lead to the overexpression of MMPs, disrupting the balance between MMPs and their inhibitors [177,178]. This results in extracellular matrix degradation and reduced mechanical properties of the ventricular wall. Carlini et al. designed an MMP-responsive hydrogel using sterically constrained cyclic peptides, which flow freely when injected and quickly form hydrogels when linearized by disease-associated MMP enzymes [65]. In vivo experiments demonstrated that the hydrogel could transition from a sol to a gel state at the MI site in rat models, enhancing cardiac function and mitigating adverse ventricular remodeling after MI.

Reactive oxygen species (ROS) signaling, including superoxide anions, H_2_O_2_, and hydroxyl radicals, is increased after MI, causing oxidative stress, inflammation, and irreversible myocardial damage [179,180]. Hence, Zheng et al. developed redox-responsive hydrogels with antioxidant properties to counter excessive ROS signaling and suppress oxidative stress-induced injury [133]. This injectable hydrogel carried liposomes containing elamipretide and sphingosine-1-phosphate as therapeutic agents. It exhibited localized delivery to damaged cardiomyocytes’ mitochondria, releasing encapsulated liposomes in a feedback-regulated manner and consuming the overproduced pathological ROS. This approach improved cardiomyocyte activity and enhanced MI treatment. Animal studies demonstrated enhanced mitochondrial function, suppressed pathological ROS production, stimulated endothelial cell tube formation, and reduced infarcted area size.

## 5. Challenges and Future Perspectives

Smart hydrogels exhibit the potential to enhance therapeutic outcomes through their sensitivity to various stimuli. Nevertheless, there are limitations and obstacles that must be overcome in order to optimize these DDSs.

One significant limitation is the safety of newly developed materials. Given the growing utilization of newly synthesized polymers and chemical components in smart hydrogel construction, it is imperative to thoroughly assess and verify their safety prior to their utilization in clinical applications. Materials that have been approved by the FDA or those with a history of prolonged use without notable side effects are preferred choices for the fabrication of stimuli-responsive hydrogels.

Another limitation is the need for novel smart hydrogel systems that exhibit enhanced and precise stimuli responses in clinical trials. Despite numerous publications on stimuli-responsive hydrogel systems, only a handful have successfully transitioned to practical clinical use [4,181,182]. The majority of published stimuli-responsive hydrogel systems are not suitable for product development. Take glucose-responsive hydrogels, for instance, where the primary issue is that most proposed systems exhibit sluggish responsiveness to fluctuations in blood-glucose levels [183]. The second issue is the insufficient efficacy of glucose-responsive hydrogels in human clinical trials. Although certain smart hydrogels, like A1 and B29-oligofucosyl-insulin (MK-2640), have yielded promising outcomes in diabetic dog and minipig models [184], the successful evaluation of glucose-responsive insulin delivery systems in human clinical trials has not yet been achieved [185]. This discrepancy is likely attributed to the incomplete understanding of quantitative differences across species, which complicates the prediction of clinical outcomes of glucose-responsive insulins when translated to humans.

Addressing these limitations is vital to advancing the field of stimuli-responsive hydrogels. Future advancements rely on expanding their capabilities through the integration of innovative biomaterials and the utilization of cutting-edge fabrication techniques. The creation of more intricate hydrogel structures, closely mimicking the natural cellular microenvironment, can be achieved by harnessing microfluidic systems or the revolutionary potential of 3D-printing technology [24,186,187,188]. This opens up exciting possibilities for engineering hydrogels that can seamlessly adapt to the dynamic microenvironment within the human body [189]. Concurrently, there is significant potential in the development of smart hydrogels with multi-stimuli responsiveness, which holds the key to achieving precision and personalization in drug delivery strategies [190,191].

## 6. Conclusions

This brief review has highlighted the recent advancements in stimuli-responsive hydrogels as smart drug delivery carriers. The intelligent responsiveness of hydrogels and nanogels to various stimuli, including pH, temperature, light, redox conditions, and biological molecules, has been comprehensively analyzed. These gels have the potential to encapsulate various kinds of therapeutic agents, spanning from traditional chemicals to biomolecular drugs (proteins, peptides, and genes), as well as cells and nanoparticles, thus establishing a broad range of applications within pharmaceutical technology. The review has also discussed their diverse applications in disease treatments and shed light on the challenges and future prospects of these smart hydrogels. The development of smart hydrogel-based DDSs holds great promise for achieving precise and personalized medicine. Overall, this review aims to give researchers a systematic understanding of smart hydrogels, while inspiring them to explore novel smart hydrogels for disease treatment.

## Figures and Tables

**Table 1:** Types of smart hydrogels and their general mechanism of action.

Type of Smart Hydrogels	Examples of Key Chemical Moieties	Mechanism of Action	References
pH-responsive	PMAAc, PDMA, anhydrides, hydrazone, imine	The hydrogel contains acidic or basic functional groups that can ionize in response to changes in pH levels, leading to swelling or shrinking of the hydrogel, and resulting in localized drug release in a site-specific manner.	[38,39,40,41,42,43,44,45,46,47,48]
Thermoresponsive	NIPAM, MBA, PEGDA	The hydrogel undergos a sol–gel phase transition in response to changes in temperature, enabling controlled drug release.	[49,50,51]
Photo-responsive	Arylazopyrazoles, o-nitrobenzyl ester, azobenzene, black phosphorous	The hydrogel contains photosensitive moieties capable of undergoing reversible or irreversible photoisomerization or photothermal reactions upon light exposure. These reactions lead to alterations in the hydrogel’s physical or chemical properties, enabling the achievement of step-by-step drug release, one-time drug release, or gradual drug release from the hydrogel.	[52,53,54,55,56,57,58,59]
Redox-responsive	GSH selenide group	The hydrogel contains redox-active groups that can undergo reversible oxidation or reduction in response to changes in redox conditions, leading to changes in the hydrogel’s physical or chemical properties, and can rapidly release encapsulated drugs at the target site.	[60,61,62,63,64]
Enzyme responsive	Hyaluronidase cinnamyloxy groups C-VPLS↓LYSG-C	The hydrogel contains enzymatically degradable linkages that respond to enzymatic activity in specific environments, allowing controlled therapeutic release at the target site.	[65,66,67]
Glucose responsive	Gox, PBA, Con A	The hydrogel contains GOx, PBA, or Con A, which can detect glucose levels in their surroundings, inducing insulin release in a glucose-responsive manner.	[68,69,70]

Note: ↓ symbol indicates the cleavage site.

**Table 2:** Types of diseases and their unique pathological or biological conditions used for smart hydrogel design.

Type of Diseases	Examples of Unique Pathological or Biological Conditions Used in Smart Hydrogel Design	Utilized Smart Hydrogel	References
Oral disease	The inflammation in chronic periodontitis and peri-implantitis leads to increased MMP-8 level locally	MMP-responsive hydrogel	[112]
	Oral inflammation leads to pH changes in the microenvironment	pH-responsive hydrogel	[113,114]
	The oral physiological temperature naturally exceeds the hydrogel’s LCST, which consequently triggers a sol–gel transition of the hydrogel	Thermoresponsive hydrogel	[114]
Cancer	Tumor cells often exhibit elevated quantities of MMPs and other proteolytic enzymes, which result in the disintegration of the extracellular matrix and the consequent creation of a conducive environment for tumor expansion	MMP-2-responsive hydrogel	[115]
	Tumor tissues generally possess a lower pH compared to normal tissues	pH-responsive hydrogel	[116]
	Tumor cells exhibit elevated GSH levels, as a consequence of significant tumorigenesis and inflammatory reactions	Redox-responsive hydrogel	[117]
	Tumor tissues typically display a hypoxic environment, which arises from inadequate blood vessel growth	Hypoxia-sensitive hydrogel	[118,119,120,121]
Wound repair and topical application	Chronic wounds often experience an oxygen deficiency, which can significantly impede the healing process	Redox-responsive hydrogel	[122]
	Chronic wound fluid exhibits increased radical scavenging activity and glutathione levels	Redox-responsive hydrogel	[123]
	Wound surface exhibits a decreased pH value	pH responsive hydrogel	[72]
Neurological diseases	Restoring spinal cord injuries lead to increased MMP levels within the injury microenvironment	MMP-responsive hydrogel	[124]
	The physiological temperature can induce sol–-gel transition of hydrogel	Thermoresponsive hydrogel	[125]
Diabetes	Both type I and II diabetes result in high blood-glucose levels	Glucose-responsive hydrogel	[74,101,126,127,128,129,130,131,132]
	The gastrointestinal (GI) tract is composed of various distinct regions, each characterized by its unique pH values	pH-responsive hydrogel	[74]
Cardiovascular and cerebrovascular diseases	The occurrence of myocardial infarction (MI) has the potential to result in the excessive expression of MMPs	MMP-responsive hydrogel	[65]
	Myocardial infarction leads to an increase in ROS signaling, which includes superoxide anions, H2O2, and hydroxyl radicals	Redox-responsive hydrogel	[133]

## Data Availability

Not applicable.
